# Antibiofilm Activity of LL-37 Peptide and D-Amino Acids Associated with Antibiotics Used in Regenerative Endodontics on an Ex Vivo Multispecies Biofilm Model

**DOI:** 10.3390/life12111686

**Published:** 2022-10-24

**Authors:** Ana C. C. Pereira, Alana P. S. Aguiar, Leticia M. P. Araujo, Larissa O. Dantas, Marcia P. A. Mayer, Lamprini Karygianni, Thomas Thurnheer, Ericka T. Pinheiro

**Affiliations:** 1 Department of Dentistry, School of Dentistry, University of São Paulo, São Paulo 05508-000, Brazil; 2 Department of Biomaterials and Oral Biology, School of Dentistry, University of São Paulo, São Paulo 05508-000, Brazil; 3 Department of Microbiology, Institute of Biomedical Sciences, University of São Paulo, São Paulo 05508-000, Brazil; 4 Clinic of Preventive Dentistry, Periodontology and Cariology, Center of Dental Medicine, University of Zurich, 8032 Zürich, Switzerland

**Keywords:** oral biofilms, regenerative endodontics, triple antibiotic paste, antibiofilm agents, antimicrobial peptides, D-amino acids

## Abstract

The antimicrobial peptide LL-37 and D-amino acids (D-AAs) have been proposed as antibiofilm agents. Therefore, this study aimed to test the antimicrobial effect of antibiofilm agents associated with antibiotics used in regenerative endodontic procedures (the triple antibiotic paste—TAP: ciprofloxacin + metronidazole + minocycline). An endodontic-like biofilm model grown on bovine dentin discs was used in this study. After 21-day growth, the biofilms were treated with 1 mg/mL TAP, 10 μM LL-37, an association of LL-37 + TAP, 40 mM D-AAs solution, an association of D-AAs + TAP, and phosphate-buffered saline (negative control). Colony forming unit (CFU) data were analyzed by two-way ANOVA and Tukey’s multiple comparison test (*p* < 0.05). LL-37 + TAP showed the best antibacterial activity (7-log^10^ CFU/mL ± 0.5), reaching a 1 log reduction of cells in relation to the negative control (8-log^10^ CFU/mL ± 0.7) (*p* < 0.05). In turn, no significant reduction in bacterial cells was observed with TAP, LL-37, D-AAs, and D-AAs + TAP compared to the negative control. In conclusion, the combination of antibiotics and LL-37 peptide showed mild antibacterial activity, while the combination of antibiotics and D-AAs showed no activity against complex biofilms.

## 1. Introduction

Regenerative endodontics is a treatment option for immature teeth with pulp necrosis (i.e., teeth with incomplete root formation and infected root canals) [1,2]. This approach allows for root development and increased thickness of dentin walls, thus decreasing the risk of tooth fracture [3]. Apical stem cells and growth factors play a key role in repair, while root canal infection may impact the outcome of regenerative procedures [4,5,6]. Therefore, the search for strategies that combine biocompatibility and antimicrobial efficacy represents a great challenge in regenerative endodontics.

During regenerative procedures, the use of mechanical instruments for root canal cleaning and shaping is not recommended due to the fragility of the root canal walls [1,2]. Therefore, antimicrobial substances used as irrigants and intracanal medication play a key role in regenerative endodontics. Current antimicrobial strategies for a regenerative procedure include an intracanal medication with antibiotics at low concentrations in order to minimize the cytotoxic effects on apical cells [7]. Specifically, a mixture of ciprofloxacin, metronidazole, and minocycline—called triple antibiotic paste—is commonly used to disinfect root canals in regenerative endodontics [7]. However, antibiotics may have limitations due to their antimicrobial spectrum or microbial resistance [2,8]. In addition, microbial biofilms in root canals may confer increased resistance to antibiotics, due to reduced microbial growth rates and low diffusion of drugs through the biofilm matrix [9]. Therefore, new approaches have been investigated to promote biofilm matrix disruption or microbial dispersion, thus improving antibiotic penetration and efficacy [10].

LL-37 is a human-derived broad spectrum antimicrobial peptide that is found in most bodily fluids as a component of the innate immune system [11]. Recombinant forms of LL-37 and its derivate peptides have been developed over the past decade to increase its efficacy and reduce its cost [11]. LL-37 promotes rupture of bacterial membranes, creating pores and altering their permeability, thus leading to cell lysis. Previous studies have highlighted its capacity to disrupt pre-formed biofilms of both Gram-positive and Gram-negative organisms [12,13]. Moreover, LL-37 has the capacity to neutralize lipopolysaccharides (LPS) from Gram-negative bacteria [14,15], modulate immune responses [16,17] and induce mineralized tissue formation [16,17], with low levels of cytotoxicity to human cells [11].

D-amino acids (D-AAs) have also been investigated as antibiofilm agents due to their ability to promote biofilm dispersion. Although D-alanine (D-Ala) and D-glutamate (D-Glu) are widely known as components of peptidoglycan, nonconventional D-AAs produced by bacteria can interfere with the bacterial cell wall synthesis, promoting D-Ala displacement [18,19]. Kolodkin-Gal et al. [18] described the role of D-leucine (D-leu), D-methionine (D-met), D-tyrosine (D-tyr), and D-tryptophan (D-trp) in biofilm disassembly, showing that they act on amyloid fibers that bind biofilm cells. D-amino acids have proven to be effective antibiofilm strategies against different bacterial species, including *Bacillus subtilis*, *Staphylococcus epidermidis*, *Staphylococcus aureus*, *Pseudomonas aeruginosa*, *Streptococcus mutans*, and *Enterococcus faecalis* [18,19,20,21,22,23,24]. In particular, D-leucine (D-leu) enabled the disruption of biofilms formed by *E. faecalis*, which is a pathogen commonly found in persistent endodontic infections [22]. Indeed, another study has shown that D-AAs (D-leu, D-met, D-tyr, and D-trp), in combination with antimicrobials used for root canal treatment, were able to disrupt *E. faecalis* biofilms [23].

Endodontic infections in immature teeth with pulp necrosis are usually chronic diseases caused by polymicrobial biofilms adhered to the root canal walls. Therefore, the complexity of endodontic biofilms must be considered in the search for effective antimicrobial strategies during endodontic treatment [25]. Different biofilm models have been used to evaluate the effectiveness of antimicrobial agents in endodontics. *E. faecalis* monospecies biofilms were used in most in vitro studies, while multispecies biofilm models were used less frequently [25,26]. In addition to biofilm complexity (i.e., number of species), biofilm age is also an important factor in biofilm models, as mature biofilms are different from young ones in terms of structure and response to endodontic disinfection strategies [26].

In regenerative endodontics, antibiofilm agents such as LL-37 and D-AAs may have potential use as intracanal medication in association with the triple antibiotic paste. LL-37 and D-AAs when used alone or in association with antibiotics have shown promising results in killing monospecies biofilms, especially those formed by medical pathogens [12,13,20,21,22,23,24]. However, there is no report on the killing activities of LL-37 or D-AAs in association with antibiotics against complex multispecies biofilms such as endodontic biofilms. Therefore, this study aimed to test whether the association of antibiofilm agents with antibiotics used in regenerative procedures would be an effective strategy to kill bacteria in an endodontic-like biofilm model composed of 10 bacterial species.

## 2. Materials and Methods

### 2.1. Biofilm Preparation

Biofilm preparation was performed as previously described [25,26]. The biofilm model consisted of 10 bacterial species commonly found in endodontic infections, including early biofilm colonizers (*Actinomyces oris* OMZ 745, *Enterococcus faecalis* ATCC 29212, *Streptococcus mutans* UA159, *Streptococcus oralis* SK 248, and *Veillonella dispar* ATCC 17748), the intermediate species (*Fusobacterium nucleatum* KP-F2) and the strict anaerobic species generally found as late colonizers of oral biofilms (*Parvimonas micra* ATCC 33270, *Porphyromonas gingivalis* ATCC 33277, *Prevotella intermedia* ATCC 25611, and *Selenomonas sputigena* ATCC 35185).

Initially, bovine dentin discs (dimensions of 9 mm × 4 mm × 0.5 mm) were placed in 24-well cell culture plates and conditioned in 1 mL of saliva [27]. The same batch of saliva was used in all experiments. For the preparation of this batch, unstimulated saliva was obtained 1.5 h after eating, drinking or brushing teeth. Saliva was then pasteurized at 60 °C for 30 min, centrifuged and stored at −20 °C [27]. The effectiveness of pasteurization was evaluated by plating aliquots of saliva in Columbia blood agar plates, which were incubated at 37 °C for 72 h, under both aerobic and anaerobic conditions. The discs immersed in saliva were incubated for 4 h at room temperature under agitation to allow the formation of the acquired salivary pellicle and then transferred to new 24-well cell culture plates.

For biofilm formation, all bacterial strains were initially adjusted to an optical density of 1.0 (550 nm) and then mixed in equal volumes. Next, the bacterial inoculum (200 μL of bacterial mix and 1.6 mL of mFUM medium) was added to the wells containing the discs [28]. The mFUM medium, which is a tryptone yeast-based medium supplemented with Sörensen’s buffer (67 mM) and carbohydrates (0.3% of glucose), was prepared as described previously [28]. Biofilms were incubated at 37 °C under anaerobic conditions for 21 days, with a fresh medium supplied every 48 h.

### 2.2. Biofilm Treatments

Fresh treatment solutions were prepared on the day of the experiment. The triple antibiotic solution (TAP) consisted of a mixture of ciprofloxacin, metronidazole and minocycline (Fórmula & Ação, São Paulo, SP, Brazil), at a concentration of 1 mg/mL each, as recommended by the AAE Clinical Considerations for a Regenerative Procedure [7]. Considering the balance between antibacterial activity and biocompatibility, the antimicrobial peptide LL-37 (Sigma-Aldrich, St Louis, MO, USA) was used at a final concentration of 10 μM [16]. The D-amino acid mixture consisted of 40 mM D-methionine (D-met), D-leucine (D-leu), D-tryptophan (D-trp), D-serine (D-ser), D-threonine (D-thr), D-phenylalanine (D-phe) and D-valine (D-val), and 0.8 mM D-tyrosine (D-tyr) (Sigma-Aldrich, St. Louis, MO, USA) [20]. The final concentration of each component of the associations (TAP + LL-37 and TAP + eight D-AAs) was the same as that of the isolated agent.

Before treatments, the discs were washed twice in phosphate buffered saline to remove cells not adhered to biofilms. The discs were immersed for 24 h in 1 mL of the following solutions: TAP (1 mg/mL); LL-37 (10 µM), association of LL-37 + TAP (final concentration of 10 μM and 1 mg/mL, respectively), 40 mM D-AA, association of D-AAs + TAP (final concentration of 40 mM and 1 mg/mL, respectively), and phosphate buffered saline (PBS) as a negative control group. The experiments were performed in triplicate on three different days.

### 2.3. Bacterial Quantification

After two washes in phosphate-buffered saline to remove the treatment solutions, the discs were transferred to a tube containing 1 mL of saline. To detach the biofilms from the disks, they were initially vortexed for three minutes, then sonicated at 30 W for ten seconds and briefly vortexed again. A serial dilution was performed and 50 µL of bacterial suspensions were plated onto Columbia blood agar plates supplemented with 5% defibrinated sheep blood. Then, the plates were incubated at 37 °C under anaerobic conditions. After 5 days of bacterial growth, colony forming units (CFU) were counted with the aid of a stereomicroscope. Differentiation of the species was achieved by observation of colonial morphology in conjunction with microscopic examination of cells from selected colonies.

### 2.4. Statistical Analysis

Colony forming units (CFUs) data from three independent experiments, each performed in triplicate, were analyzed using Prism statistical analysis software v.8.4.3 (GraphPad, La Jolla, CA, USA). The CFUs’ counts of the groups with antimicrobial treatments and the untreated (control) group are presented as medians (maximum and minimum values). After log transformation of cells counts, two-way ANOVA and Tukey’s multiple comparison tests were used to assess differences between groups, considering a value of *p* < 0.05 as a significant difference.

## 3. Results

**Figure 1:**
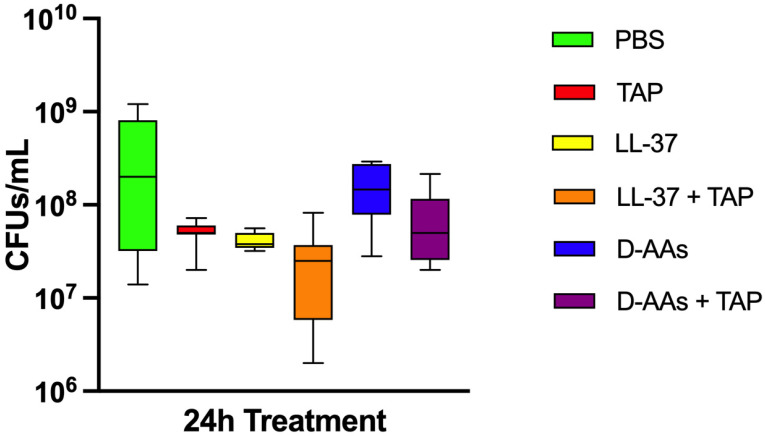
Boxplots demonstrating colony forming units’ (CFUs) counts after 24 h treatments of multispecies biofilms with phosphate-buffered saline (negative control), mixture of ciprofloxacin + metronidazole + minocycline (TAP), LL-37 antimicrobial peptide, association of LL-37 + TAP, mixture of 8 D-amino acids (D-AAs) and association of D-AAs + TAP. The medians of CFUs after treatments are shown on the inner lines, while the whiskers indicate the minimum and maximum values.

Figure 1 shows colony forming units’ (CFUs) counts in biofilms treated with PBS (negative control), mixture of ciprofloxacin + metronidazole + minocycline (TAP), LL-37, LL-37 + TAP, and a mixture of eight D-AAs and 8 D-AAs + TAP. No significant reduction in bacterial cells was observed with TAP (8-log^10^ CFUs/mL ± 0.2), LL-37 (8-log^10^ CFUs/mL ± 0.09), D-AAs (8-log^10^ CFUs/mL ± 0.3) and D-AAs + TAP (8-log^10^ CFUs/mL ± 0.4) compared to the negative control (8-log^10^ CFUs/mL ± 0.7). In turn, the treatment of biofilms with LL-37 + TAP (7-log^10^ CFUs/mL ± 0.5) showed the best antibacterial activity, reaching a 1-log reduction in cells in relation to the control (*p* = 0, 0013). However, no difference was found when comparing TAP treatment alone and the combinations with either LL-37 or D-AAs (*p* > 0.05).

## 4. Discussion

This study investigated the effect of an antibiotic mixture (metronidazole + ciprofloxacin + minocycline) in association with antibiofilm strategies on a multispecies endodontic-like biofilm. Antibiotics in combination with the antimicrobial peptide LL-37 promoted bacterial reduction, while antibiotics alone showed no significant reduction compared to the negative control. Likewise, previous studies have shown the limited activity of antibiotics against biofilms, mainly due to their poor penetration across the biofilm matrix [8,9]. More importantly, our findings point to a potential synergism between the antimicrobial peptide LL-37 and antibiotics commonly used in regenerative endodontics. Antimicrobial peptides have been used to promote biofilm disruption, increasing the diffusion of antibiotics and thereby enhancing their antimicrobial activity [11,29]. For instance, a previous study showed that LL-37 derivate peptides enhanced the effect of chloramphenicol and ciprofloxacin against *Staphlylococcus aureus* and *Escherichia coli* biofilms [29].

Pulp necrosis and consequent root canal infection in immature teeth prevent complete root development. The purpose of regenerative endodontic procedures is to disinfect root canals and preserve the viability of periodontal tissue stem cells, providing favorable conditions for root development and/or strengthening [1,2]. Microorganisms are usually found as polymicrobial biofilms adhered to the root canal walls of immature teeth with pulp necrosis. From a clinical point of view, an important feature of microbial biofilms is their tolerance to antimicrobial agents [9]. Resistance may be related to several factors, including restricted penetration of antimicrobials through the biofilm matrix, bacterial diversity in biofilms, and the metabolic state of bacterial cells [9]. For example, dormant cells in biofilms are generally unaffected by antibiotics that act on replicating cells [9]. In the present study, antibiotics commonly used for regenerative procedures (ciprofloxacin, metronidazole and minocycline) were not effective against multispecies biofilms. These data confirmed a previous report showing that triple antibiotic paste (TAP) at low concentrations (1 mg/mL) is not able to kill biofilm cells [30]. A recent review pointed to the need to explore alternative approaches to TAP, such as antibiotic-releasing nanofibers, delivery vehicles, and distinct antibiotic formulations [8].

The development of biofilm control strategies represent future prospects for treatment of complex polymicrobial infections, including the use of substances that prevent bacterial adhesion, interrupt microbial cells communication, or promote biofilm disruption [10]. In this context, the present study showed that the inclusion of antimicrobial peptides in antibiotic formulations may represent a strategy to be further explored. Several microbiology studies on medical pathogens described the synergy between antimicrobial peptides that interact with the bacterial membrane and antibiotics that inhibit the synthesis of nucleic acids (e.g., metronidazole and ciprofloxacin) as well as proteins (e.g., minocycline) [11]. Combinations of LL-37 and antibiotics have shown synergistic effects against diverse medical pathogens, including *Pseudomonas aeruginosa*, *Staphylococcus aureus*, *Enterococcus faecium*, *Enterococcus faecalis*, *Clostridioides difficile*, *Klebsiella pneumonaie*, *Acinetobacter baumannii*, *Stenotrophomonas maltophilia*, *Micrococcus luteus*, and *Streptococcus* spp. [11]. In the present study, LL-37 associated with antibiotics was the best antimicrobial strategy tested, suggesting that LL-37 may have facilitated the entry of antibiotics into biofilms where they could exert antimicrobial effects. However, the effect of the association tested against multispecies biofilms was still very limited, which points to the need to investigate other concentrations of both antimicrobial peptides and antibiotics to identify those that are synergistic.

Ideally, intracanal medication in regenerative endodontic procedures should be used in bactericidal concentrations, with minimal effects on cell viability of apical periodontal tissue, which is responsible for apical repair [1,2]. In this context, LL-37 has potential use in regenerative endodontics due to its low levels of cytotoxicity and its ability to disrupt biofilms [12,13]. Its ability to neutralize lipopolysaccharides [14,15] also represents an important feature, as the endodontic microbiota is mainly composed of Gram-negative anaerobic bacteria. Furthermore, the ability of LL-37 to induce hard tissue formation may contribute to apical repair and root apex closure in immature teeth with pulp necrosis [16,17]. In the present study, LL-37 concentration was chosen based on previous studies evaluating its biocompatibility and antibiofilm activity. At concentrations similar to or below 10 μM, LL-37 showed no cytotoxic effect on eukaryotic cells [17] and was able to kill biofilm cells or disrupt established biofilms [13]. This antibiofilm activity may be particularly important for regenerative procedures because microbial biofilms may remain untouched in un-instrumented root canals [31]. Although the mechanisms of its anti-biofilm activities are not fully understood, studies have shown that LL-37 is able to decrease the binding of bacteria to each other or to surfaces, disrupting intracellular quorum sensing molecules and promoting twitching motility [11]. Furthermore, after penetrating biofilms, LL-37 can kill bacteria by rupturing their cell membranes [32,33,34]. A previous study showed that LL-37 was effective against *Staphylococcus aureus* biofilms, achieving bacterial reduction of more than 4 logs [13]. In contrast, treatment with LL-37 alone was not effective against the endodontic biofilm model used in the present study. Such discrepancies may be due to differences in biofilm models, since multispecies biofilms are expected to be more resistant than single-species biofilms [35]. In addition, biofilm maturation (21 days) in the present study may also have been a contributing factor to increased bacterial resistance [26].

Another antibiofilm strategy tested in this study was the use of D-amino acids (D-met, D-leu, D-tyr, D-trp, D-ser, D-thr, D-phe, and D-val) to promote biofilm dispersion and thus enhance antimicrobials activity. As dispersed cells are expected to be more susceptible to antimicrobials than biofilms, D-AAs have been investigated as adjuvants to antibiotics in the treatment of biofilms. D-amino acids have been tested as antibiofilm strategies against pathogens such as *Staphylococcus aureus*, *Pseudomonas aeruginosa*, *Enterococcus faecalis*, and *Escherichia coli*, proving to be effective in inhibiting biofilm formation or dispersing preformed biofilms. Moreover, the use of D-AAs has increased the activity of several antibiotics [21,24]. For instance, there was a synergistic effect between D-AAs (D-asp and D-glu) and ciprofloxacin against *Staphylococcus aureus* and *Pseudomonas aeruginosa* biofilms [21,24]. This association has been explored as a promising strategy to overcome the antimicrobial resistance of the aforementioned medical pathogens. In contrast, in the present study, no bacterial reduction was observed after treatment of biofilms with D-AAs alone or in combination with antibiotics. Divergences between studies may be due to differences in study models, including bacterial biofilm composition, selected antibiotics, and bacterial susceptibility.

Unlike other diseases, no specific pathogen is associated with endodontic infections, which are caused by nonspecific polymicrobial communities organized as intraradicular biofilms [36,37]. Therefore, this complex community in the root canals is highly resistant to antimicrobial strategies. In this context, it is important to emphasize the need to use a biofilm model that best represents the richness and diversity of endodontic infections [38]. The model used in the present study included ten endodontic pathogens, with strict and facultative anaerobic species, Gram-positive and Gram-negative: *Actinomyces oris*, *Enterococcus faecalis*, *Fusobacterium nucleatum*, *Parvimonas micra*, *Porphyromonas gingivalis*, *Prevotella intermedia*, *Selenomonas sputigena*, *Streptococcus oralis*, *Streptococcus mutans*, and *Veillonella dispar*. The antimicrobial resistance of this biofilm may have been increased by the presence of *Enterococcus faecalis*. This endodontic-like biofilm model was previously validated by Lukic et al. [25] and represents a modification of a well-established in vitro multispecies biofilm model [39,40]. Despite its limitations (e.g., lack of anatomical complexity of root canals and direct contact with antimicrobials), this biofilm model provides a resilient community-response pattern. Other advantages of this biofilm model include standardization and reproducibility. Future studies should be directed to identify the residual bacteria after antimicrobial treatment of biofilms.

## 5. Conclusions

In conclusion, the combination of the antimicrobial peptide LL-37 and antibiotics commonly used for regenerative endodontic procedures (metronidazole, ciprofloxacin, and minocycline) showed mild antibacterial activity, while antibiotics, D-amino acids, or a combination of both showed no antibacterial activity against an endodontic-like biofilm model. These data reinforce that antibiotics at clinical concentrations recommended for regenerative endodontic procedures are ineffective against complex biofilms. Therefore, new antimicrobial strategies should be investigated for treatment of immature teeth with pulp necrosis. In this context, the association of antibiotics and antimicrobial peptides deserves further investigation since they showed a potential antimicrobial synergism. In addition, future studies should investigate other biological properties of this association, including its cytotoxicity and ability to induce the formation of mineralized tissues, as they may impact the outcome of regenerative endodontic procedures.

## Data Availability

Data is contained within the article.
